# Supplementary material for: Adolescent perspectives on peripartum mental health prevention and promotion from Kenya: Findings from a design thinking approach
Source: PLoS One. 2024 Jan 2;19(1):e0290868. doi: 10.1371/journal.pone.0290868 (PMC10760697; doi:10.1371/journal.pone.0290868)
Supplement: S1 Table — (DOCX) [file pone.0290868.s001.docx]

**Supplement Table 1: Key Informant Interview Guide**

| Interview domains   1. ***Interview guide for pregnant adolescents***   **Barriers**  Who all are in your family/who do you live with currently/schooling- how are you being treated by schoolmates/friends?   1. How does pregnancy experience seem from the point of view of a pregnant adolescent? 2. How does the pregnancy come about? 3. How does the adolescent come to reconcile – given that many despite being thrown into unplanned pregnancy don’t say it is unwanted? 4. How is your relationship with your parent/caregiver now that you are pregnant? 5. What sort of mismatch happens around emotional attachment to a partner and understanding practically about their support in their lives. 6. How does problem solving happen around the following barriers?  - Social - Emotional & relationship - Practical & economic  1. What worries do you have around the delivery of the baby? 2. How is pregnancy in early stages vs later stages understood?   **Solutions-**   1. How do you keep yourself from getting another unplanned pregnancy and falling into problems further? Who helps you on that and who all can help better? 2. What is the role of emotional and mental health in that pregnancy experience? 3. When you become stressed what do you do to come out of it? 4. What gives you hope during this time? 5. What sort of friends can help or have helped you so far? 6. What kinds of things do you think will become easier as time goes by?   **UCD/HCD based**   1. How do others view you? What kind of criticism do you receive from others? 2. What is your mother/father/sibling/school/teacher/friend thinking of you? 3. Where and how do they understand or misunderstand what you are going through? 4. How would you like to be seen? 5. What do you do on a daily basis? 6. How would you wish things to be? 7. Who helps you feel better? 8. Who helps you communicate with your parents/caregivers/friends? 9. What did you like doing before pregnancy and what is it that you stopped doing? 10. ***Interview guide for adolescent mothers***   **Barriers**   1. How does pregnancy and motherhood experience seem from the point of view of a young mother? 2. What do you come to understand your personal and family relationships as you have become a mother now? 3. How does the adolescent come to reconcile – given that many despite being thrown into unplanned pregnancy don’t say it is unwanted – how is that experience like given you have a little baby to care for? 4. What kind of support do you receive from your partner? 5. How does problem solving happen around the following barriers?  - Social - Emotional & relationship - Practical & economic  1. What are your worries around care or health or wellbeing of the baby? 2. What are the things that have become tougher/difficult now that you have given birth? 3. How was life a year back and now? 4. What things would you wish to change? 5. Who are the people who are helping you now? 6. Who are the people that helped you during your pregnancy? 7. What do you do for entertainment?   **Solutions-**   1. How do you keep yourself from getting another unplanned pregnancy and falling into problems further? Who helps you on that and who all can help better? 2. What is the role of emotional and mental health in that pregnancy experience? 3. When you become stressed what do you do to come out of it? 4. What gives you hope during this time? 5. What sort of friends can help or have helped you so far? 6. What kinds of things do you think will become easier as time goes by and now that you have a new baby?   **UCD/HCD based**   1. How do others view you? 2. What sort of relationship do you have with your baby? 3. How does it change or evolve with time? 4. What is your mother/father/sibling/school/teacher/friend thinking of you as a mother/ young mother? 5. Where and how do they understand or misunderstand you? 6. How would you like to be seen? 7. What do you do on a daily basis? 8. How would you wish things to be? 9. Who helps you feel better? 10. Who helps you communicate with your parents/caregivers/friends? 11. What did you like doing before motherhood and what is it that you stopped doing? |
| --- |
