# Supplementary material for: Adolescent perspectives on peripartum mental health prevention and promotion from Kenya: Findings from a design thinking approach
Source: PLoS One. 2024 Jan 2;19(1):e0290868. doi: 10.1371/journal.pone.0290868 (PMC10760697; doi:10.1371/journal.pone.0290868)
Supplement: Attachment — Submitted filename: comments.docx [file pone.0290868.s002.docx]

Manuscript Number: PONE-D-22-21939

Title: Adolescent perspectives on peripartum mental health prevention and promotion from
Kenya: Findings from a design thinking approach

**General comment**

**T**he paper addresses an important topic because adolescent mental health vulnerabilities during pregnancy and the postpartum period are critical. Because it focuses on a specific population of adolescents in Kenya, this manuscript makes an important contribution to the literature.  Below are some suggestions for improving the manuscript's quality.

**Abstract and title**

- Title and abstract cover the content of the work.
- Authors can remove references from the abstract part
- It is also preferable if the abstract sections written in independent paragraphs

**Introduction**

- The introduction is well written. However, the following suggestions will improve the paper
- The authors should clearly state the research gap that they presume to fill. A more explicit description in a few lines would definitely help.
- The objectives described in the manuscript look like those of the main project other than specific to this study. It would be better to clearly state the objectives of this study.

Method

- In general the method part is well written and detail

 Analysis: I am not sure on the robustness of the analysis method

Result: Generally good

**Discussion** looks good

**Conclusion**: Good

**Ethical concern**: There are no ethical concerns for this paper

**Competing interest:** I have no competing interest
