# Supplementary material for: Adolescent perspectives on peripartum mental health prevention and promotion from Kenya: Findings from a design thinking approach
Source: PLoS One. 2024 Jan 2;19(1):e0290868. doi: 10.1371/journal.pone.0290868 (PMC10760697; doi:10.1371/journal.pone.0290868)
Supplement: Attachment — Submitted filename: Revieweresponse.docx [file pone.0290868.s003.docx]

Response to the reviewers	8^th^ November 2022

PONE-D-22-21939
Adolescent perspectives on peripartum mental health prevention and promotion from Kenya: Findings from a design thinking approach
PLOS ONE

Dear Editor

We thank you for you feedback and review of the above mentioned paper and please find attached a

- A rebuttal letter that responds to each point raised by the academic editor and reviewer(s).
- A marked-up copy of manuscript that highlights changes made to the original version.
- An unmarked version of revised paper without tracked changes.

Response to the editor requests

1. Please ensure that your manuscript meets PLOS ONE's style requirements, including those for file naming. The PLOS ONE style templates can be found at

https://journals.plos.org/plosone/s/file?id=wjVg/PLOSOne_formatting_sample_main_body.pdf and

<https://journals.plos.org/plosone/s/file?id=ba62/PLOSOne_formatting_sample_title_authors_affiliations.pdf>

*response: we have now provided all the relevant information here.*

2. Please provide additional details regarding participant consent. In the ethics statement in the Methods and online submission information, please ensure that you have specified what type you obtained (for instance, written or verbal, and if verbal, how it was documented and witnessed). If your study included minors, state whether you obtained consent from parents or guardians. If the need for consent was waived by the ethics committee, please include this information.

*Response: we sought written consent from participants and this is documented on page 10.*

3. Thank you for stating the following financial disclosure:

“The funders had no role in study design, data collection and analysis, decision to publish, or preparation of the manuscript.”

At this time, please address the following queries:

a)        Please clarify the sources of funding (financial or material support) for your study. List the grants or organizations that supported your study, including funding received from your institution.

b)        State what role the funders took in the study. If the funders had no role in your study, please state: “The funders had no role in study design, data collection and analysis, decision to publish, or preparation of the manuscript.”

c)        If any authors received a salary from any of your funders, please state which authors and which funders.

d)        If you did not receive any funding for this study, please state: “The authors received no specific funding for this work.”

Please include your amended statements within your cover letter; we will change the online submission form on your behalf.

*Response:* *a)* *the main body of the work was embedded in a NIH/ Fogarty International Centre funded study to the senior author. However key activities of HAT were funded by UNICEF’s Innocenti centre in a program called ‘Helping Adolescents Thrive’. B) some of the program managers of UNICEF were part of a decision process around codesigning interventions that addresses the global research gap around pregnant, parenting and general population of adolescents, in that spirit they participated in the scientific enterprise but they did not interfere or decided next steps for the team. c) Part of Malia Duffy’s time was supported by UNICEF. No team member received financial remuneration for developing this manuscript.*

4. We note that you have indicated that data from this study are available upon request. PLOS only allows data to be available upon request if there are legal or ethical restrictions on sharing data publicly. For more information on unacceptable data access restrictions, please see http://journals.plos.org/plosone/s/data-availability#loc-unacceptable-data-access-restrictions.

In your revised cover letter, please address the following prompts:

  a) If there are ethical or legal restrictions on sharing a de-identified data set, please explain them in detail (e.g., data contain potentially sensitive information, data are owned by a third-party organization, etc.) and who has imposed them (e.g., an ethics committee). Please also provide contact information for a data access committee, ethics committee, or other institutional body to which data requests may be sent.

   b) If there are no restrictions, please upload the minimal anonymized data set necessary to replicate your study findings as either Supporting Information files or to a stable, public repository and provide us with the relevant URLs, DOIs, or accession numbers. For a list of acceptable repositories, please see http://journals.plos.org/plosone/s/data-availability#loc-recommended-repositories.

We will update your Data Availability statement on your behalf to reflect the information you provide.

*Response: there are some restrictions on the use of de-identified data to protect the participants who are young people and provided sensitive information. the data can be shared upon reasonable request from the corresponding author.*

5. We note that you have stated that you will provide repository information for your data at acceptance. Should your manuscript be accepted for publication, we will hold it until you provide the relevant accession numbers or DOIs necessary to access your data. If you wish to make changes to your Data Availability statement, please describe these changes in your cover letter and we will update your Data Availability statement to reflect the information you provide.

*Response: We are suggesting that the data would be available upon reasonable request from the corresponding author.*

6. Your ethics statement should only appear in the Methods section of your manuscript. If your ethics statement is written in any section besides the Methods, please delete it from any other section.

*The ethics statement only appears in the Methods section.*

7. Please ensure that you refer to Figure 1 and 2 in your text as, if accepted, production will need this reference to link the reader to the figure.8. We note you have included a table to which you do not refer in the text of your manuscript. Please ensure that you refer to Tables 2 and 4 in your text; if accepted, production will need this reference to link the reader to the Table.*Response for questions 7 and 8: we have checked the placement of the ethics statement, referred to figure 1 and 3 in the text as well as included tables 2 and 4 in the text. See pages XXXX*

 Response to the reviewer comments

Reviewer #1: Manuscript Number: PONE-D-22-21939

Title: Adolescent perspectives on peripartum mental health prevention and promotion from
Kenya: Findings from a design thinking approach
General comment
The paper addresses an important topic because adolescent mental health vulnerabilities during pregnancy and the postpartum period are critical. Because it focuses on a specific population of adolescents in Kenya, this manuscript makes an important contribution to the literature. Below are some suggestions for improving the manuscript's quality.

*Response: we thank the reviewer for their kind comments and have tried to address their comments to the best possible.*

Abstract and title
• Title and abstract cover the content of the work.
• Authors can remove references from the abstract part
• It is also preferable if the abstract sections written in independent paragraphs

*Response: we have now removed the references and have reorganized the references*

Introduction
• The introduction is well written. However, the following suggestions will improve the paper
• The authors should clearly state the research gap that they presume to fill. A more explicit description in a few lines would definitely help.
• The objectives described in the manuscript look like those of the main project other than specific to this study. It would be better to clearly state the objectives of this study.

*Response: we have now made the research gap that the paper tries to address a bit clearer. We have also tried to restate the study objectives. The reviewer is right to say that those do emanate from a broader study but we have tried to present them in an academic manner needed for a publication.*

Method
• In general the method part is well written and detail

*Response: we thank you for your positive feedback.*

Analysis: I am not sure on the robustness of the analysis method

*Response: we have tried to be transparent about our intentions to treat this paper in an adolescent centered manner*

Result: Generally good

*Response: we thank you for these comments and positive feedback*
Discussion looks good
Conclusion: Good
Ethical concern: There are no ethical concerns for this paper

*Response: we thank you for these comments*
