# Supplementary material for: Adolescent perspectives on peripartum mental health prevention and promotion from Kenya: Findings from a design thinking approach
Source: PLoS One. 2024 Jan 2;19(1):e0290868. doi: 10.1371/journal.pone.0290868 (PMC10760697; doi:10.1371/journal.pone.0290868)
Supplement: Attachment — Submitted filename: Reviews.docx [file pone.0290868.s004.docx]

**Adolescent perspectives on peripartum mental health prevention and promotion from Kenya: Findings from a design thinking approach - COMMENTS**

This manuscript reports a gap in adapting interventions for peripartum adolescents. However, the theory of Change workshop i.e. advisory group discussions with FGDs, journals etc should be a combined manuscript to avoid salami slicing. If available, including data from young fathers could help in strengthening the manuscript. The manuscript also seems to address “adolescent perspectives on peripartum mental health prevention and promotion from Kenya” yet a lot of information is provided on the advisory group. Although it was the same study, it is not clear how this information is responding to the proposed AIMs. Otherwise, the title can change to reflect this.

1. Remove references from the abstract.
2. Check for no spacing, double punctuation marks, spaces etc throughout the manuscript
3. While this statement is true” Global prevalence studies demonstrate a wide variation in estimates of postpartum depression among adolescents, ranging between 14-53%”, it is not clear how it is linked to the statement before and after. The introduction needs to flow.
4. Page 5: “A recent program evaluation in Kenya”……describes an existing program, yet the statement that follows “In the context of Kenya however, further understanding factors from the…” contradicts this earlier statement and the factors to be explored are not stated. The aim of the proposed study is also not clearly stated.
5. The design thinking process needs to be explained and its rationale discussed in the manuscript – perhaps at the introduction section.

**Methods:**

1. Elaborate on the advisory groups? How was data collected? How many FGDs were conducted? How many participants were in each FGD? Is this what the author’s refer to as Theory of Change workshops? The way the methods section is structured is a bit confusing to the reader.
2. There was an advisory board consisting of various stakeholders yet only discussions with pregnant women and policy makers were conducted and reported separately. Was there a rationale for this?
3. 17-22 key informant interviews seem a high number for a homogenous group of pregnant mothers. What was the rationale for this number?
4. A WhatsApp-based group is only introduced in the results section rather than the methods section.
